# Biological Functions of Silver Nanowires in Inhibiting *Vibrio* Pathogens and Modulating Shrimp Hemocyte Immunity

**DOI:** 10.3390/life16040545

**Published:** 2026-03-26

**Authors:** Smruti R. Sahoo, Zhen-Hao Liao, Fan-Hua Nan

**Affiliations:** Department of Aquaculture, National Taiwan Ocean University, Keelung City 202301, Taiwan; smrowty@gmail.com

**Keywords:** silver nanowires, anti-*Vibrio* activity, immunomodulatory, shrimp hemocytes

## Abstract

Silver nanoparticle (AgNP)-based products have been increasingly applied in aquaculture due to their antimicrobial properties and capacity to modulate host immunity. This study investigated the biological activities of synthesized silver nanowires (AgNWs), with particular emphasis on their anti-*Vibrio* efficacy and immunomodulatory effects, to evaluate their potential application in shrimp aquaculture. Antibacterial activity was assessed using nonlinear regression analysis to determine minimum inhibitory concentrations (MICs) against three major *Vibrio* pathogens, while cytotoxicity and immune responses were evaluated using white shrimp hemocytes through cell viability assays and in vitro gene expression analysis, respectively. AgNWs exhibited antibacterial effects on *Vibrio parahaemolyticus*, *Vibrio alginolyticus*, and *Vibrio harveyi*, with MIC values of 873.7, 58.78, and 672.1 μg/mL, respectively. Hemocyte viability remained above 90% at AgNW concentrations of up to 1000 mg/L, indicating good biocompatibility. AgNWs significantly upregulated immune-related lipopolysaccharide and β-1,3-glucan-binding protein (*LGBP*) and *Toll* gene expression at specific concentrations, indicating immunostimulation. These results suggest that AgNWs possess antibacterial activity and immunomodulatory potential with low cytotoxicity, supporting their promise as a novel functional agent for shrimp disease management.

## 1. Introduction

*Vibrio* species, including *Vibrio alginolyticus*, *Vibrio harveyi*, and *Vibrio parahaemolyticus*, are major opportunistic pathogens that cause substantial economic losses in aquaculture due to mass mortality events in cultured species [1]. To mitigate infection risks in aquaculture systems, antibiotics are commonly administered; however, their extensive and often unregulated use has contributed to the emergence of multidrug-resistant (MDR) *Vibrio* strains [2,3]. *Vibrio* species exhibit a high degree of genetic plasticity and can readily acquire antimicrobial resistance genes through mobile genetic elements such as plasmids, integrons, and transposons [4]. This adaptability enables them to rapidly respond to selective pressures, particularly those arising from the frequent use of antibiotics in aquaculture environments [5]. Moreover, *Vibrio* spp. can persist across diverse aquatic ecosystems and throughout the seafood production chain, thereby increasing the risk of human exposure to MDR strains [6]. This situation is further exacerbated by their ability to form biofilms, which not only protect them from harsh environmental conditions but also enhance their resistance to antibiotics and disinfectants commonly used in aquaculture practices [7,8].

Given the escalating challenge of controlling MDR *Vibrio* strains, increasing attention has been directed toward alternative antimicrobial strategies capable of overcoming the limitations of conventional antibiotics. Among these approaches, silver nanoparticles (AgNPs) have emerged as a promising option due to their potent and multifaceted antibacterial mechanisms [9]. Unlike many antibiotics that act on specific molecular targets, AgNPs exert antimicrobial effects through multiple simultaneous pathways. Reactive oxygen species (ROS) generated by AgNPs can directly damage bacterial cell membranes, disrupt DNA replication, and inhibit essential enzymatic activities, thereby accelerating bacterial inactivation and death [10]. Recent studies have demonstrated that both chemically synthesized and green-synthesized AgNPs (produced using microorganisms, plant extracts, or biomolecules) are highly effective against major *Vibrio* pathogens [11,12] and various MDR strains in vitro and in vivo [13,14]. The antimicrobial activity extends beyond planktonic cells to include the inhibition and disruption of biofilm formation, a critical virulence factor that enhances *Vibrio* persistence, environmental resilience, and resistance to antimicrobial agents in marine ecosystems [15]. Collectively, these properties position AgNPs as promising candidates for next-generation antimicrobial strategies aimed at safeguarding public health and promoting the sustainability of aquaculture practices [16].

Several AgNP-based products have been applied in aquaculture disease management due to their antimicrobial properties and their ability to enhance the immune status of aquatic animals when administered at optimal doses and appropriate timing [17,18]. In the case of aqueous exposure to AgNPs, previous studies have demonstrated their therapeutic and protective potential in *Oreochromis niloticus* against *Aeromonas hydrophila* infection. Exposure to 0.8 mg/L of AgNPs for 7 days was shown to mitigate immune suppression, bacterial colonization, cytotoxicity, oxidative stress, inflammatory responses, and tissue damage [19]. Similarly, treatment with 10 μg/L of AgNPs for 8 weeks improved growth performance, enhanced non-specific immune responses and antioxidant enzyme activities, and alleviated the histopathological effects of *A. hydrophila* challenge in the gills, dermis, liver, and intestine [20]. Previous studies have reported that dietary AgNPs can activate various immune responses to enhance resistance against shrimp pathogen infections. For example, *Prosopis chilensis*-synthesized AgNPs administered to *Penaeus monodon* increased total hemocyte counts and phenoloxidase activity, thereby providing a protective effect against Vibriosis [21]. *Emblica officinalis-* or *Ficus racemosa*-synthesized AgNPs administered to *P. monodon* enhanced the expression of immune-related genes, including interferon regulatory factor (*IRF*), viral antigen gene (*Vago*), prophenoloxidase (*proPO*), and penaeidin, thereby providing protective effects against *V. parahaemolyticus* infection [22]. The efficacy of *Chaetomorpha antennina*-synthesized AgNPs in enhancing disease resistance against *Vibrio harveyi* and protecting against tissue damage has been demonstrated through oral administration in *Macrobrachium rosenbergii* [23].

Despite extensive research on spherical AgNPs, increasing attention has recently been directed toward anisotropic silver nanostructures, particularly silver nanowires (AgNWs) [24], which may exhibit distinct physicochemical and biological properties [25,26]. AgNWs possess unique structural features, including high aspect ratios, enhanced surface contact with bacterial membranes, and improved penetration into biofilms, which contribute to their biological activities. These advantages have been well documented in studies involving bacterial species such as *Staphylococcus aureus* and *Escherichia coli* [27]. More broadly, the biological activities of nanosilver are strongly influenced by its physicochemical characteristics, including particle size at the nanoscale, shape, dispersion state, and colloidal stability [28]. Moreover, the application of nanosilver requires rigorous safety evaluations, including assessments of optimal dosage and material stability [29]. However, information regarding the application of AgNPs or AgNWs in shrimp aquaculture remains limited. Therefore, the present study aims to investigate the biological activities of the synthesized AgNWs, with particular emphasis on their in vitro anti-*Vibrio* activity, immunomodulatory effects, and biosafety, in order to evaluate their potential applications in shrimp aquaculture.

## 2. Materials and Methods

### 2.1. Synthesis of AgNWs

Silver nitrate (AgNO_3_; ≥99.9%; Sigma-Aldrich, St. Louis, MO, USA), polyvinylpyrrolidone (PVP; Mw ≈ 55,000; Sigma-Aldrich, St. Louis, MO, USA), copper (II) chloride dihydrate (CuCl_2_·2H_2_O; ≥99.0%; Sigma-Aldrich, St. Louis, MO, USA), and ethylene glycol (EG; anhydrous, ≥99%; J.T. Baker, Phillipsburg, NJ, USA) were used without further purification. Deionized water with a resistivity of 18.2 MΩ·cm was used throughout the synthesis process. The synthesis of anisotropic silver nanowires was carried out using a typical polyol process employing ethylene glycol [30]. In a standard procedure, 20 mL of EG was preheated in a 50 mL three-neck round-bottom flask fitted with a reflux condenser, magnetic stirrer, and thermometer to ensure consistent temperature and mixing. The reaction mixture was heated to 160 °C under continuous stirring in a closed system to minimize evaporation and maintain thermal stability. Meanwhile, three precursor solutions were prepared separately in EG: AgNO_3_ at a concentration of 94 mM, PVP at 0.3 mM, and copper (II) at 4 mM. Once the temperature of EG reached a stable 160 °C, 80 µL of the CuCl_2_-EG solution was added to the hot EG. Next, the AgNO_3_-EG solution was loaded into a syringe and injected into the reaction flask at 160 °C at a flow rate of 0.4 mL/min over a period of 15 min under continuous stirring. A different syringe was then used to inject the freshly prepared PVP-EG solution into the heated mixture at 160 °C at 0.4 mL/min for 15 min. During this process, the reaction mixture gradually turned from colorless to light gray and then deepened in color, indicating nucleation and growth of silver nanostructures. Following complete addition of the precursors, the mixture was maintained at 160 °C for an additional 90 min to ensure complete reduction of the silver ions and to promote the growth of well-defined nanowires. Following the reaction, the solution was allowed to cool naturally to room temperature. The resulting AgNW-containing suspension was centrifuged at 3000× *g* for 30 min to collect the nanowires as a pellet at the bottom of the centrifuge tube. The supernatant was carefully discarded, and the pellet was re-dispersed in deionized water. This purification process was repeated several times to remove unreacted precursors and byproducts.

### 2.2. Characterization of AgNWs

The morphology of the as-synthesized silver nanowires (AgNWs) was initially examined using an optical microscope (Olympus, Tokyo, Japan) equipped with a 40× objective lens to assess their general shape and dispersion. Transmission electron microscopy (TEM) was also utilized to examine the diameter and smoothness of the as-synthesized AgNWs. The colloidal AgNW suspension was drop-cast onto copper-coated TEM grids and allowed to air-dry under ambient conditions. The dried grids were then loaded into a HT-7700 transmission electron microscope (Hitachi, Tokyo, Japan), operated at an accelerating voltage of 80 kV, to obtain detailed images of the nanowires. Additionally, the optical properties of the AgNWs were characterized using UV–Visible (UV–Vis) spectroscopy (Molecular Devices, San Jose, CA, USA).

### 2.3. Antibacterial Properties of AgNWs

The *Vibrio* pathogens used in the present study were kindly provided by Dr. Po-Tsang Lee (Department of Aquaculture, National Taiwan Ocean University). *V. parahaemolyticus*, *V. alginolyticus*, and *V. harveyi* stocks were cultured overnight on tryptic soy agar (TSA; TITAN BIOTECH, Bhiwadi, India). Single colonies were then inoculated into tryptic soy broth (TSB; TITAN BIOTECH, Bhiwadi, India) and grown overnight at 30 °C. The overnight cultures were subsequently subcultured into fresh TSB and incubated until they reached the log phase (OD_600_ = 0.47, 0.53, and 0.50 for *V. parahaemolyticus*, *V. alginolyticus*, and *V. harveyi*, respectively), corresponding to a bacterial concentration of approximately 10^7^ CFU/mL. The antibacterial activities against each strain (10^3^ CFU/mL) were assessed by incubating the bacteria in triplicate with serial concentrations of AgNWs (0, 5, 10, 25, 50, 100, 250, 500, 1000, and 2000 μg/mL) for 1 h in Phosphate-Buffered Saline (PBS; GENESTAR, Kaohsiung, Taiwan), followed by their inoculation onto TSA plates for 24 h. Average colony numbers were recorded, and the minimum inhibitory concentration (MIC) values were determined using nonlinear regression analysis in GraphPad software Prism, version 10 (Boston, MA, USA) [31].

### 2.4. In Vitro Shrimp Hemocyte Cytotoxicity Toward AgNWs

Hemocytes isolated from white shrimps (*Penaeus vannamei*) were mixed with anticoagulant buffer (450 mM NaCl, 10 mM KCl, 10 mM HEPES, and 10 mM EDTA-Na_2_ at pH 7.3) in a ratio of 1:9. The hemocyte pellets were then collected by centrifugation at 800× *g* for 20 min at 4 °C and resuspended in modified complete Hank’s Balanced Salt Solution (MCHBSS; HBSS (Gibco, Waltham, MA, USA) supplemented with 10 mM CaCl_2_, 3 mM MgCl_2_, and 5 mM MgSO_4_). The resulting cell suspensions were adjusted to 3 × 10^6^ cells/mL for cytotoxicity assays and 1 × 10^7^ cells/mL for immunity tests. All chemicals used for buffer preparation were obtained from Sigma-Aldrich (St. Louis, MO, USA).

In the cytotoxicity assay, 100 μL of the hemocyte suspension was dispensed into a clear, flat-bottom, untreated 96-well plate (Simply, Taoyuan, Taiwan) and centrifuged at 800× *g* for 10 min at 4 °C to remove the anticoagulant buffer. The cells were then incubated with MCHBSS containing various concentrations of AgNWs (0–1000 mg/L) for 30 min. The incubations were performed in triplicate. After incubation, the solutions were removed, and 50 μg of MTT reagent (3-(4,5-dimethylthiazol-2-yl)-2,5-diphenyltetrazolium bromide; Sigma-Aldrich, St. Louis, MO, USA) was added to each well and incubated for 4 h at 37 °C. The medium was subsequently removed, and dimethyl sulfoxide (Gibco, Waltham, MA, USA) was added to dissolve the formazan crystals. Absorbance was measured at 570 nm using a microplate reader (Molecular Devices, San Jose, CA, USA). Cell viability (expressed as a percentage) was determined by calculating the ratio of the treated group’s absorbance to the control group’s absorbance and then multiplying it by 100. Cytotoxic effects were evaluated by determining changes in cell viability.

### 2.5. In Vitro Shrimp Hemocyte Immune and Stress Responses Toward AgNWs

For the immunity assays, a 1 mL aliquot of the hemocyte suspension was centrifuged at 800× *g* for 10 min at 4 °C. The cell pellet was resuspended and incubated with 1 mL of MCHBSS (control) or MCHBSS containing AgNWs (10, 100, and 1000 mg/L) for 30 min. The incubations were performed in triplicate. After incubation, the buffer was removed by centrifugation. Total RNA was extracted using GENEzol reagent (Geneaid, New Taipei, Taiwan) strictly following the manufacturer’s instructions, including sequential steps of tissue lysis, phase separation, RNA precipitation, washing, and solubilization. Subsequently, first-strand cDNA was synthesized from 1 μg of total RNA using the MIIIs™ 1st Strand cDNA Synthesis Kit (Bionovas, Toronto, ON, Canada), also according to the manufacturer’s protocol, including residual genomic DNA removal and First-Strand cDNA synthesis. The expression levels of immune-related genes (including prophenoloxidase I (*proPO I*), superoxide dismutase (*SOD*), glutathione peroxidase (*GPx*), lysozyme (*Lyz*), anti-lipopolysaccharide factor (*ALF*), lipopolysaccharide and β-1,3-glucan-binding protein (*LGBP*), and Toll-like receptor (*Toll*)), stress-related genes (including heat shock protein 70 (*Hsp70*) and metallothionein (*MT*)), and apoptosis-related genes, including *Caspase 3* and B-cell lymphoma 2 (*Bcl2*)), were quantified by quantitative PCR (QuantStudio 1, Applied Biosystems, Waltham, MA, USA) using SYBR Green-based detection (AMPLIQON, Odense, Denmark) with previously reported primers [32,33,34]. The thermal cycling conditions were 95 °C for 15 min, followed by 40 cycles of 95 °C for 15 s and 60 °C for 1 min. Relative gene expression was calculated using the 2^−^^ΔΔCT^ method [35] and normalized to the expression level of elongation factor 1α.

### 2.6. Statistical Analyses

Statistical analyses were performed using SPSS software version 22.0 (IBM Corp., Armonk, NY, USA). The Shapiro–Wilk test and Levene’s test were used to assess the normality of data distribution and homogeneity of variances, respectively. One-way ANOVA followed by Tukey’s honest significant difference (HSD) post hoc test was applied to determine statistical differences among treatments, with the significance level set to *p* < 0.05.

## 3. Results

### 3.1. Characterization of AgNWs

**Figure 1:**
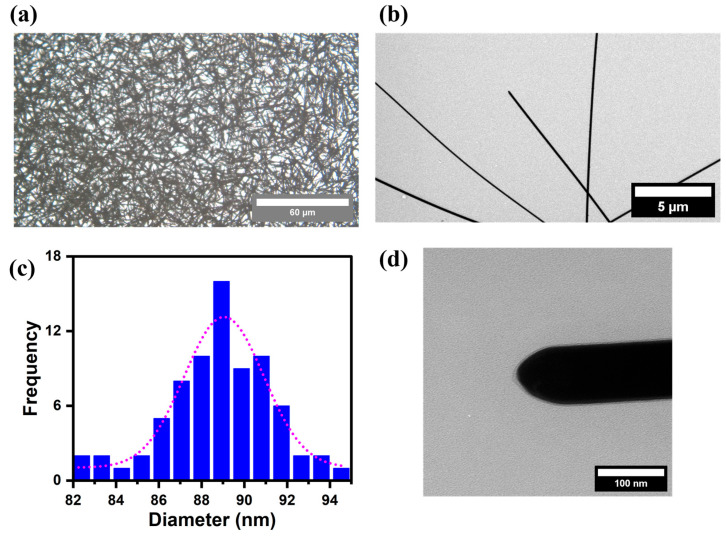
Characterization of silver nanowires (AgNWs). (**a**) Observation under an optical micro-scope (Scale Bar: 60 mm), (**b**) representative TEM image of AgNWs (Scale Bar: 5 mm), (**c**) size distribution of the AgNW diameters and (**d**) magnified image of the AgNWs as observed in TEM (Scale Bar: 100 nm).

**Figure 2:**
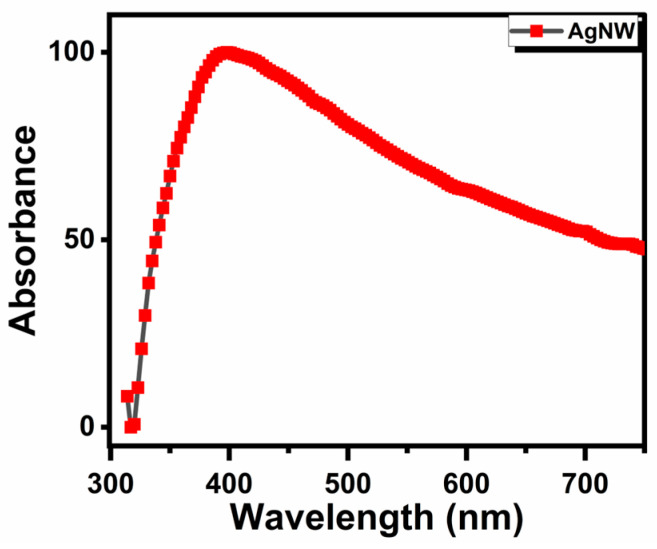
The optical absorbance spectrum of the as-synthesized AgNWs in aqueous medium. It exhibits a broad absorbance feature extending from the visible to the near-infrared (NIR) region. This broad spectrum corresponds to the longitudinal mode of surface plasmons as well as light scattering effects arising from the AgNWs. The peaks observed in the near-UV/blue region was attributed to the transverse mode of surface plasmons arising from the AgNWs.

Figure 1 displays the morphologies of the as-synthesized AgNWs, as observed under an optical microscope and a transmission electron microscope. The 40× optical micrograph (Figure 1a) reveals that the AgNWs consisted of elongated cylindrical structures arranged in a randomly crossed wire configuration. The average length of the AgNWs was estimated from the optical micrographs and was found to be approximately 40–50 μm (Figure 1b). Although the network appeared as randomly crossed AgNWs, three predominant configurations were observed: single AgNWs, two aligned AgNWs, and crossed AgNWs. The diameters exhibited a narrow size distribution (Figure 1c), with an average diameter of approximately 90 nm. In addition, the nanowires displayed sharp edges and smooth surface morphology, as shown in Figure 1d. Figure 2 shows the optical absorbance spectrum from the as-synthesized AgNWs. The localized surface plasmon resonance (LSPR) peak can be observed at around 350–400 nm, corresponding to the transverse plasmon mode. As shown in Table S1, the zeta potential measurement indicates moderate stability of AgNWs in deionized water, with an absolute value greater than 10 mV, suggesting a low tendency to agglomerate.

### 3.2. Antibacterial Effects of AgNWs on Vibrio Pathogens

**Figure 3:**
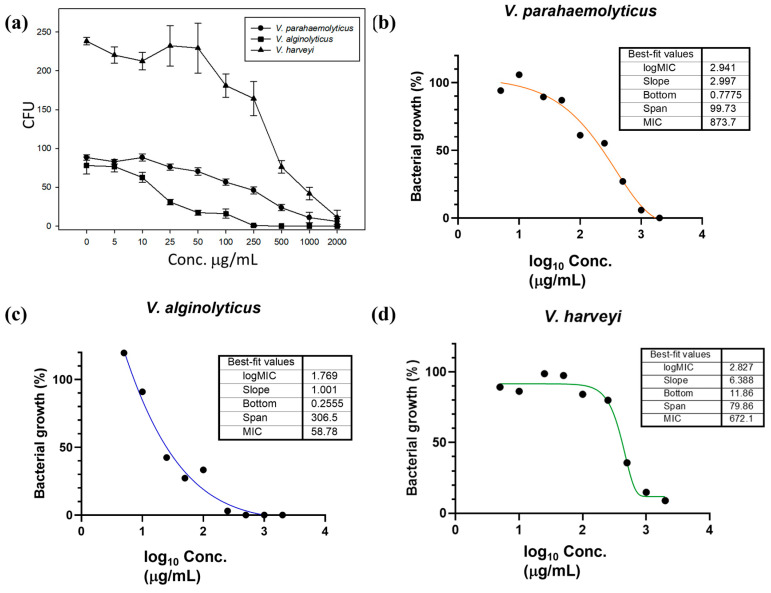
(**a**) Growth inhibition of *Vibrio* spp. after one hour of exposure to AgNWs in PBS. Values are shown as means ± SDs (*n* = 3). Minimum inhibitory concentration (MIC) determination for (**b**) *V. parahaemolyticus*, (**c**) *V. alginolyticus*, and (**d**) *V. harveyi* using nonlinear regression analysis.

The antibacterial efficacy of AgNWs was evaluated using a nonlinear regression method. As shown in Figure 3a, the growth of the three *Vibrio* pathogens was progressively inhibited with the increase in AgNW concentrations. AgNWs exhibited antibacterial activity against *V. parahaemolyticus* (Figure 3b), *V. alginolyticus* (Figure 3c), and *V. harveyi*, (Figure 3d) with MIC values of 873.7, 58.78, and 672.1 μg/mL, respectively.

### 3.3. In Vitro Effects of AgNWs on Cell Viability and Immunity

**Figure 4:**
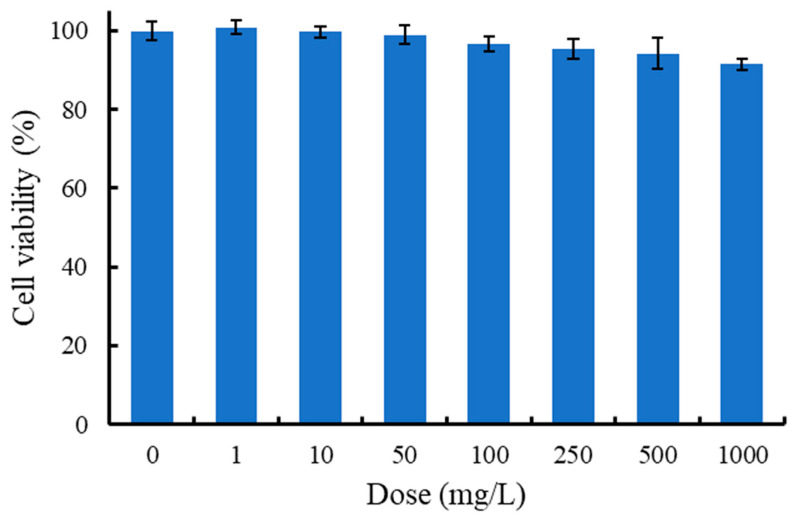
Cell viability (%) in the hemocytes of *P. vannamei* after in vitro incubation with 0 (control) to 1000 mg/L of AgNWs for 30 min. Values are shown as means ± SDs (*n* = 3).

**Figure 5:**
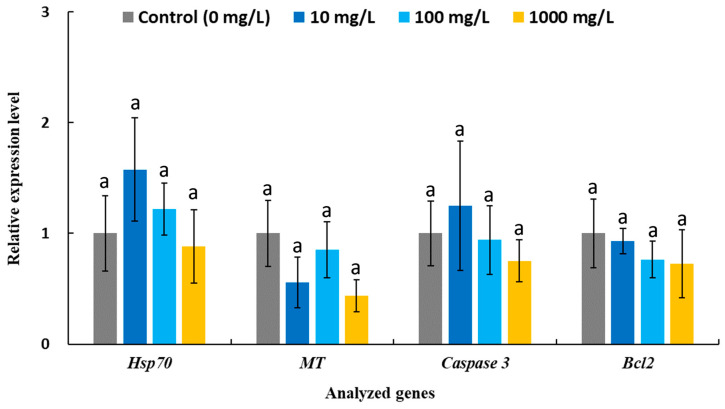
The relative expressions of stress- or apoptosis-related genes in the hemocytes of *P. vannamei* after in vitro incubation with 0 (control) and indicated concentrations of AgNWs for 30 min. Values are shown as means ± SDs (*n* = 3). One-way ANOVA and Tukey’s test were used for analysis. Treatments sharing the same letters above the bars indicate no significant differences (*p* > 0.05) for each analyzed gene.

**Figure 6:**
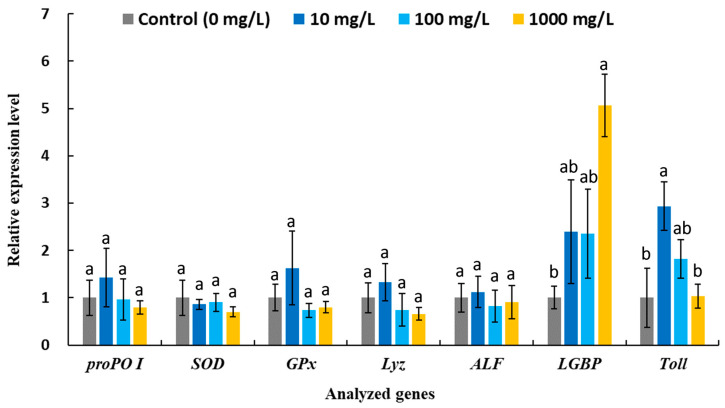
The relative expressions of immune-related genes in the hemocytes of *P. vannamei* after in vitro incubation with 0 (control) and indicated concentrations of AgNWs for 30 min. Values are shown as the mean ± SD (*n* = 3). One-way ANOVA and Tukey’s test were used for analysis. Significant differences (*p* < 0.05) between treatments for each analyzed gene are indicated by the different letters above the bars.

The cytotoxicity of AgNWs was evaluated using shrimp hemocytes. As shown in Figure 4, the MTT assay results indicate that the AgNWs exhibited good biocompatibility, with cell viability remaining above 90% at treatment concentrations of up to 1000 mg/L. Moreover, the expression levels of stress- and apoptosis-related genes in hemocytes were not significantly altered following in vitro exposure to AgNWs (10, 100, and 1000 mg/L), further supporting the biocompatibility of AgNWs (Figure 5). Figure 6 shows the immune-related gene expression profiles of shrimp hemocytes treated with AgNWs. Compared with the untreated control group, in vitro AgNWs exposure did not significantly alter the expression of *proPO I*, *SOD*, *GPx*, *Lyz*, or *ALF*. However, AgNW treatment significantly upregulated *LGBP* expression at 1000 mg/L and *Toll* expression at 10 mg/L.

## 4. Discussion

A polyol method employing PVP as a capping agent was used to synthesize smooth and elongated silver nanowires [36]. In recent years, the shrimp farming industry has been severely impacted by bacterial diseases, particularly those caused by *V. parahaemolyticus*, *V. alginolyticus*, and *V. harveyi*, leading to substantial economic losses [37]. The AgNWs synthesized in this study, similar to previously reported green-synthesized AgNPs [38,39], demonstrated the ability to inhibit the growth of multiple *Vibrio* species. Previous studies have indicated that the antibacterial activity of AgNPs varies among different *Vibrio* species, with reported differences being up to approximately 4-fold. The MIC values of *Portieria hornemannii*-synthesized AgNPs were 3.9, 7.81, 7.81, and 15.62 μg/mL against *Vibrio anguillarum*, *V. parahaemolyticus*, *V. harveyi*, and *Vibrio vulnificus*, respectively [38]. Similarly, *Haliotis discus* polysaccharide–protein complex-synthesized AgNPs exhibited MIC values of 3.125, 6.25, 6.25, 12.5, and 12.5 μg/mL against *Vibrio furnissii*, *Vibrio mimicus*, *Vibrio hollisae*, *Vibrio fluvialis*, and *V. vulnificus*, respectively [39]. In contrast, the present study demonstrated substantially greater variability for AgNWs, with differences exceeding 10-fold. As demonstrated by theoretical electromagnetic field simulations (Figure S1), unlike spherical AgNPs, which exhibit localized surface plasmon, AgNWs support propagating surface plasmon modes [36]. Consequently, AgNWs facilitate multi-point interactions along the nanowire axis, in contrast to the single-point interactions characteristic of AgNPs. In addition to shape and structural factors, electric field distribution also influences the interaction between AgNPs/AgNWs and bacterial cells. The propagating electric field pattern of AgNWs suggests that interactions between bacteria and AgNWs are more complex than those occurring between bacterial cells and AgNPs. The complex interactions associated with AgNWs may contribute to the observed variation in our antibacterial activity experiments among different *Vibrio* species. Further investigations are necessary to elucidate the interactions between AgNWs and *Vibrio* cell surface structures using advanced microscopy techniques and biochemical analyses in bacteria (e.g., ROS generation, lactate dehydrogenase activity and silver accumulation). In addition, the roles of extracellular polymeric substance (EPS) production and other secreted compounds among different *Vibrio* spp. that may contribute to nanosilver resistance should be further clarified [40,41]. Interestingly, *V. alginolyticus* generally exhibited greater sensitivity to antibiotics, whereas *V. parahaemolyticus* showed comparatively higher resistance [42]. A similar pattern of susceptibility was also observed for AgNW treatment.

On the other hand, because AgNPs can induce significant cytotoxic and genotoxic effects once accumulated in cells and disrupt biological systems, determining the balance between antimicrobial efficacy and potential toxicity is a critical consideration for their practical applications [43,44]. In the present study, AgNWs exhibited negligible cytotoxic, apoptotic, and stress-related effects on shrimp hemocytes, which serve as a functional cell model for the in vitro screening of toxicological and immunomodulatory responses [45]. AgNPs of different sizes and shapes can be recognized and internalized by immune cells via distinct cellular uptake pathways [46]. The wire-like morphology of AgNWs may reduce cytotoxic effects by delaying hemocyte internalization, whereas smaller, spherical nanoparticles can interact more rapidly with cells, potentially leading to higher cytotoxicity [47,48]. Nevertheless, this hypothesis requires further experimental validation. Toxicity in aquatic environments is difficult to assess because AgNPs undergo complex transformations that alter their speciation, including dissolution, aggregation, and chemical complexation. In particular, the toxic effects observed for small nanoparticles (≤25 nm) are most likely attributable to the intrinsic toxicity of the nanoparticles themselves rather than secondary transformation products [49]. Previous studies have also shown that spherical AgNPs can induce toxic effects in both target and non-target aquatic organisms and readily accumulate in tissues even at low concentrations and following short-term exposure [50,51]. Considering shape effects, ion release behavior, and accumulation sites, AgNWs may present a lower toxicological risk to aquatic animals compared with AgNPs. The LC_50_ values of AgNPs for fish, *Daphnia*, and algae were approximately 2.3-, 11.5-, and 3.5-fold lower, respectively, than those of AgNWs [52], although the lower antibacterial activity of AgNWs compared with AgNPs is likely due to their lower specific surface area available for contacting with bacterial cells [53]. 

AgNWs possess a structural advantage when fabricated into films, as their wire-like morphology minimizes agglomeration [27]. Therefore, the use of AgNWs for antimicrobial strategies and water treatment warrants further development, particularly for integration into water filtration systems for practical use in aquaculture. For example, the immobilization of AgNPs on TEPA/Den-SiO_2_ as a water filtration medium for bacterial disinfection has been shown to improve the survival rate and growth performance of *P. vannamei* postlarvae cultured in seawater [54]. Our bacterial suspension filtration experiment demonstrated that AgNW-integrated membranes can effectively reduce *V. parahaemolyticus* levels, achieving up to a 213-fold reduction compared with untreated membranes during seawater cycling (Figure S2).

Previous studies have reported that AgNP administration can upregulate the ex-pression of various immune-related genes to enhance resistance against shrimp pathogen infections [55]. In our previous work, nanoclay-supported AgNPs significantly increased the expression of the antioxidant enzymes *SOD* and *GPx*, as well as the antimicrobial peptide *Lyz*, in hemocytes isolated from white shrimp following 7 days of dietary supplementation (2 g/kg) [45], suggesting enhanced antioxidant capacity and innate immune responses through the regulation of ROS and antimicrobial activity. Moreover, in white shrimp fed Bacillus-biomastered AgNPs (1 g/kg) for 15 days, the expression of *proPO* (involved in melanin synthesis), *ALF* (antimicrobial peptide) and *LGBP* was upregulated following *V. parahaemolyticus* challenge [56]. Notably, a previous study demonstrated that *LGBP* upregulation is a key factor contributing to increased survival in white shrimp treated with AgNPs (1.2 ng per shrimp) following infection with white spot syndrome virus (WSSV) [57]. LGBP functions as a pattern-recognition protein that recognizes pathogen-associated molecular patterns, such as β-1,3-glucan, and subsequently activates immune responses including melanin synthesis and antimicrobial peptide production [58]. In the present study, AgNWs triggered distinct immune pathways in a dose-dependent manner, as evidenced by significant upregulation of *LGBP* at 1000 mg/L and *Toll* at 10 mg/L. Activation of the Toll signaling pathway is known to induce the expression of downstream antimicrobial peptide genes involved in host defense responses, including *Lyz* and *ALF* [59]. These findings suggest that AgNWs may exert differential immuno-modulatory effects depending on exposure concentration.

Our previous in vitro studies demonstrated that nanoclay-supported AgNP treatment at different concentrations significantly enhanced multiple immune parameters in white shrimp hemocytes, including phenoloxidase activity, superoxide anion production, and phagocytic activity, with the highest levels observed at 10, 10, and 0.1 mg/L, respectively [45]. Importantly, distinct metallic nanoparticle treatment concentrations and frequency appeared to activate different immunomodulatory pathways [60]. In a previous study investigating dietary supplementation of gold nanoparticles in white shrimp, significantly higher expression levels of *Toll* were observed after 24 h of treatment in the high-dose (20 mg/kg) and medium-dose (2 mg/kg) groups compared with the low-dose (0.2 mg/kg) group. In contrast, the low-dose group exhibited significantly higher *proPO* expression than the medium- and high-dose groups [61]. This phenomenon may be closely associated with dose-dependent alterations in intracellular ROS levels [62], which in turn induce diverse immune response patterns. Nevertheless, further investigations are required to elucidate and validate the underlying immunomodulatory mechanisms of AgNWs, particularly through integrated comparisons between in vitro and in vivo immune responses. Previous studies have reported that AgNPs can modulate the expression of genes involved in inflammation, apoptosis, cell cycle regulation, and ROS defense, as well as general stress responses, potentially exerting adverse effects on exposed aquatic organisms [63]. Notably, a previous study reported that concentrations effective in inhibiting *V. alginolyticus* may also suppress immune responses in fish, highlighting potential trade-offs between antimicrobial efficacy and host immune function [11]. In our in vitro experiments, none of the immune-related genes exhibited downregulation, indicating that AgNWs possess good biocompatibility and immunological safety, thereby highlighting their potential applicability in dietary supplement and *Vibrio* disease prevention.

## 5. Conclusions

The silver nanowires (AgNWs) synthesized in this study demonstrated good biocompatibility with white shrimp hemocytes, exhibiting minimal cytotoxicity. Their low toxicity highlights the potential of AgNWs for application in sustainable aquaculture to reduce the substantial economic losses caused by *Vibrio*-associated shrimp diseases. AgNWs moderately inhibited the growth of *V. parahaemolyticus*, *V. alginolyticus*, and *V. harveyi* and enhanced non-specific immune responses in white shrimp hemocytes, suggesting potential dual antibacterial and immunomodulatory functionality. Overall, the present findings provide foundational evidence supporting the potential application of AgNWs in shrimp disease prevention and control. Future research focusing on system design and combination treatment strategies with other antimicrobial agents will be critical to sustaining the antimicrobial efficacy of AgNWs and facilitate their practical application in water treatment and veterinary applications.

## Data Availability

The data supporting the findings of this study are available from the corresponding authors upon reasonable request.

## Supplementary Materials

The following supporting information can be downloaded at: https://www.mdpi.com/article/10.3390/life16040545/s1, Figure S1. Theoretical electromagnetic field simulations of AgNWs and spherical AgNPs, Figure S2. Filtration-based antibacterial assay of silver nanowires, Table S1. Zeta potential and hydrodynamic diameter of AgNWs.
