# Supplementary material for: Biological Functions of Silver Nanowires in Inhibiting Vibrio Pathogens and Modulating Shrimp Hemocyte Immunity
Source: Life (Basel). 2026 Mar 26;16(4):545. doi: 10.3390/life16040545 (PMC13117146; doi:10.3390/life16040545)

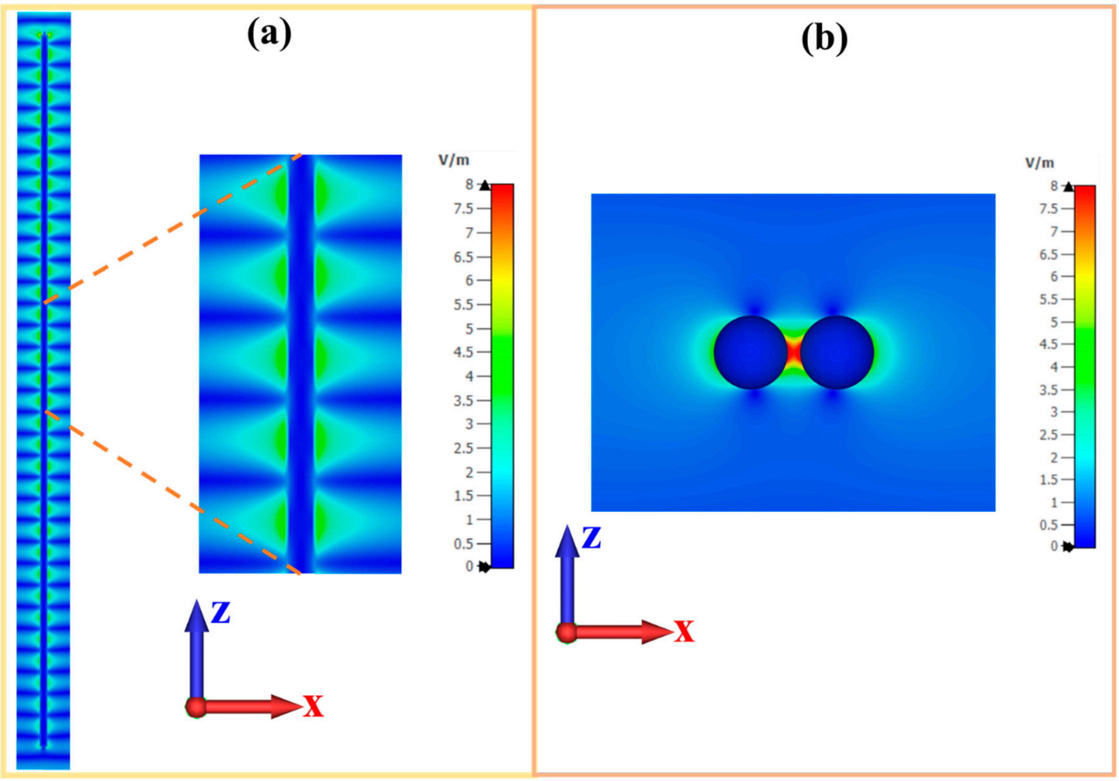

Figure S1. Theoretical electromagnetic field simulations of AgNWs and spherical AgNPs.

(a) The simulated AgNWs had a length of 9 μm and a diameter of 90 nm. (b) The simulated AgNPs had a diameter of 30 nm, with an interparticle separation distance of 5 nm. The coordinate axes and electric field scale bars are shown. A node–antinode standing wave pattern of electromagnetic fields (propagating) is observed along the longitudinal axis of the AgNWs, whereas the localized enhancement in intense electric fields is evident in the gap region between two adjacent nanospheres.

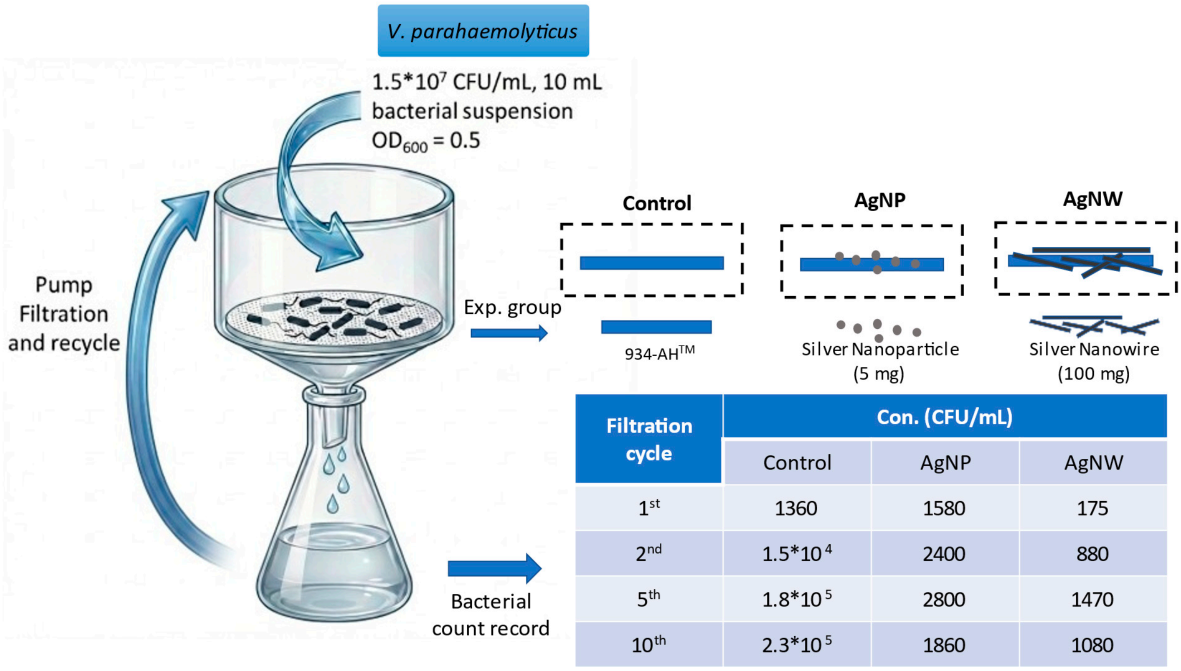

Filtration cycle	Con. (CFU/mL)		
	Control	AgNP	AgNW
1st	1360	1580	175
2nd	$1.5*10^4$	2400	880
5th	$1.8*10^5$	2800	1470
10th	$2.3*10^5$	1860	1080

Figure S2. Filtration-based antibacterial assay of silver nanowires. *Vibrio parahaemolyticus* was cultured, harvested, and resuspended in sterile seawater for subsequent experiments. Three types of filtration membranes were prepared, including a control group (control), a silver nanoparticle group (AgNP), and a silver nanowire group (AgNW). The silver nanoparticles were obtained from a commercial product (Yu Song Technology Co., Ltd.), whereas the silver nanowires were synthesized in the present study. The nano-materials were loaded onto filtration membranes (934-AH™) and allowed to air-dry. Following filtration, the *V. parahaemolyticus* suspension was plated for colony enumeration, and the filtration process was repeated for ten consecutive cycles.

Table S1. Zeta potential and hydrodynamic diameter of AgNWs.

Zeta Potential	Z-Average (d.nm)	Polydispersity Index (PDI)
-13.80 ± 1.06	222.07 ± 3.16	0.300 ± 0.01

Representative results from one replicate are shown below.

**File Name:**	20250829.dts	**Dispersant Name:**	Water
**Record Number:**	3	**Dispersant RI:**	1.330
**Date and Time:**	2025年8月29日 上午 10:29:39	**Viscosity (cP):**	0.8279
		**Dispersant Dielectric Constant:**	77.5

System

**Temperature (°C):**	28.0	**Zeta Runs:**	12
**Count Rate (kcps):**	277.8	**Measurement Position (mm):**	2.00
**Cell Description:**	Clear disposable zeta c...	**Attenuator:**	8

Results

			Mean (mV)	Area (%)	St Dev (mV)
**Zeta Potential (mV):**	-15.0	**Peak 1:**	-15.0	100.0	5.01
**Zeta Deviation (mV):**	5.01	**Peak 2:**	0.00	0.0	0.00
**Conductivity (mS/cm):**	0.0838	**Peak 3:**	0.00	0.0	0.00
**Result quality**	Good				

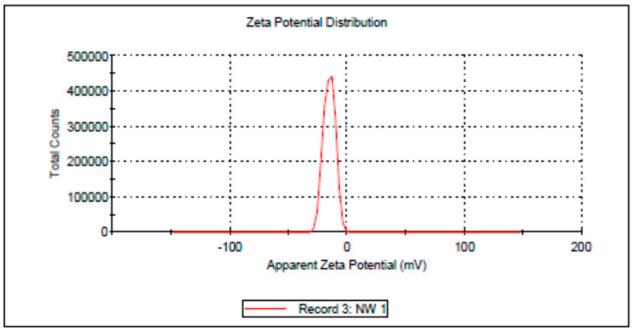

System

**Temperature (°C):**	28.0	**Duration Used (s):**	60
**Count Rate (kcps):**	259.2	**Measurement Position (mm):**	5.50
**Cell Description:**	Clear disposable zeta cell	**Attenuator:**	4

Results

			Size (d.n...	% Intensity:	St Dev (d.n...
**Z-Average (d.nm):**	218.5	**Peak 1:**	204.3	95.5	59.64
**PdI:**	0.288	**Peak 2:**	5183	4.5	483.7
**Intercept:**	0.850	**Peak 3:**	0.000	0.0	0.000
**Result quality**	Good				

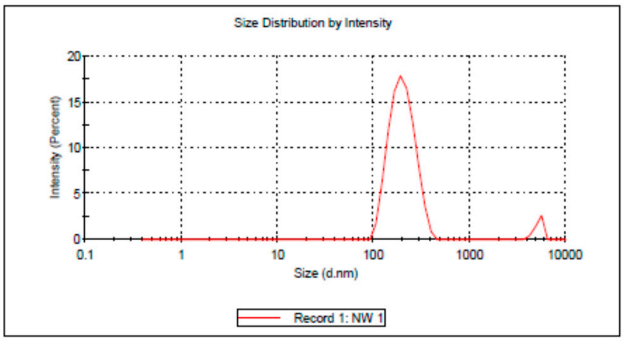

Supplement: Supplementary file 1 [file life-16-00545-s001.zip › life-4196842-supplementary.pdf]
